# Using Tannic-Acid-Based Complex to Modify Polyacrylonitrile Hollow Fiber Membrane for Efficient Oil-In-Water Separation

**DOI:** 10.3390/membranes13030351

**Published:** 2023-03-18

**Authors:** Micah Belle Marie Yap Ang, Wei-Lin Hsu, You-Syuan Wang, Hsin-Yu Kuo, Hui-An Tsai, Kueir-Rarn Lee

**Affiliations:** R&D Center for Membrane Technology, Department of Chemical Engineering, Chung Yuan Christian University, Taoyuan 32023, Taiwan

**Keywords:** hollow fiber, polyacrylonitrile, oil–water separation, membrane

## Abstract

Separating oil from water allows us to reuse both fluids for various applications, leading to a more economical process. Membrane separation has been evidenced as a cost-effective process for wastewater treatment. A hollow fiber membrane made of polyacrylonitrile (PAN) is an excellent choice for separating oil from water because of its superior chemical resistance. Its low antifouling ability, however, reduces the effectiveness of its separation. Hence, in this study, we used tannic acid (TA) and FeIII complex to modify the surface of the PAN hollow fiber membrane. To improve membrane performance, different reaction times were investigated. The results demonstrate that even when the TA-FeIII covered the pores of the PAN membrane, the water flux remained constant. However, when an emulsion was fed to the feed solution, the flux increased from 50 to 66 LMH, indicating low oil adhesion on the surface of the modified membrane. When compared to the pristine membrane, the modified membrane had superior antifouling and reusability. As a result, the hydrophilic TA-FeIII complex on PAN surface improves overall membrane performance.

## 1. Introduction

Oily wastewater is harmful to environment and humans, and its separation from water is challenging [1,2]. This waste is present in different industries such as the refinery, metallurgical, leather, pharmaceutical, textile, and food and beverage industries. Municipal wastewater also has much waste oil, especially cooking oil. Every year, the production of oily wastewater increases, especially from oil refineries [2]. Among the different oils that the world currently uses, diesel fuel is a widespread contaminant. It is usually a light, refined petroleum product that evaporates and disperses naturally if spilled in small amounts. In contrast, marine diesel fuel—used by maritime vessels—is heavier and persists longer when spilled. Both fuels can affect the marine ecosystem with small spills. Hence, there is a need for an efficient and effective method to separate it from water up to trace levels.

Membrane separations are usually applied in the secondary treatment of oil emulsions. In the primary step, mechanical processes isolate the free-floating oils from water and emulsified oils. Then, membranes are used to separate the stable emulsified oils from water. Membrane pore size can be tailored, thus, its ability to separate small and large oil droplets makes it a favorable choice. Apparently, the advantage of integrating a membrane in the separation system would be lower capital cost and the absence of chemical additives [3].

In wastewater treatment, pressure-driven membranes are applied to treat wastewater [4]. These cover microfiltration, ultrafiltration, nanofiltration, reverse osmosis and forward osmosis. Either microfiltration or ultrafiltration is suitable in treating oil-in-water emulsion [5,6,7]. Ultrafiltration requires higher pressure than microfiltration, but it can separate small oil droplets. Numerous polymers have been applied in oil–water emulsion separation, such as poly(vinylidene fluoride), poly(arylene ether nitrile), polycaprolactone, polylactic acid, poly(vinyl alcohol), poly(vinyl chloride), polyurethane and polyacrylonitrile (PAN) [8]. PAN is one of the most intensively used materials because it has good chemical resistance, especially in oils. However, its antifouling property needs to improve for efficient oil–water separation.

There are several methods to modify the polymeric membrane: embedding nanoparticles [9,10,11,12] or surface modification [13,14,15,16,17]. Embedding nanoparticles on the polymeric matrix can only give a minimal improvement in surface hydrophilicity. Surface modification with proper material would increase the hydrophilicity to a greater extent. There are several hydrophilic polymers that have been used for surface coating: poly(vinyl alcohol) [18], polydopamine [14,19], sericin [20], alginate [21] and tannic acid (TA) [22,23]. Compared with other hydrophilic polymers, the TA-FeIII complex has better antifouling and bio-inspired adhesive capability [24,25].

TA is considered a mixture of polygalloyl glucose molecules with the empirical formula of C_76_H_52_O_46_. It possesses an ability to adhere onto various kinds of substrates (organic and inorganic), even hydrophilic and hydrophobic surfaces, as well as particles and planar ones, because it contains both hydrophobic and hydrophilic parts that are responsible for interaction at different types of substrates. Usually, its hydroxyl groups interact with hydrophilic materials through hydrogen bonding [26]. Hence, it can cover the PAN surface uniformly when varying only the reaction time through hydrogen bonding interaction. Wang et al. [27] coated PVDF using TA-aminopropyltriethoxysilane(APTES)-FeIII, and the resultant membrane was reported to have an outstanding antifouling and excellent oil–water separation. Liu et al. [28] enhanced the antibacterial and algal inhibition of an ultrafiltration membrane through combining a TA-copper-iron coating. A TA-FeIII coating with sodium periodate to assist in deposition using inkjet printing technology was explored by Xie et al. [23]. However, the printing technology was only applicable for a flat sheet membrane. Having a hollow fiber configuration has evidenced efficiency for the process.

In this work, a PAN hollow fiber membrane was fabricated with poly(vinylpyrrolidone (PVP). TA-FeIII complex was deposited on the PAN surface and simultaneously improved the surface hydrophilicity and antifouling property. Changing the reaction time of TA with FeIII could improve the membrane separation efficiency. Membranes were characterized to examine their physicochemical properties and correlate them with membrane performance.

## 2. Materials and Methods

### 2.1. Materials

The PAN powder was obtained from Tong-Hwa Synthesis Fiber Co., Ltd. (Taipei, Taiwan). The PAN solvent was a reagent grade N-Methyl-2-pyrrolidone (NMP) procured from Tedia Company Inc. (Fairfield, OH, USA). PVP, MW = 1,300,000 g/mol and tannic acid were purchased from Alfa Aesar (Haverhill, MA, USA). Sodium hypochlorite was obtained from Nihon Shiyaku Industries Ltd. (Tokyo, Japan). Sodium hydroxide (NaOH), Sodium dodecyl sulfate (SDS), and iron(III) chloride anhydrous 97% EP were supplied by Showa Chemical Co., Ltd. (Tokyo, Japan). Glycerin was bought from Mingtai Chemical Co., Ltd. (Taoyuan, Taiwan). The diesel used for separation experiments was provided by PetroChina Co., Ltd. (Taipei, Taiwan).

### 2.2. Characterization

The chemical composition of the membrane surface was analyzed using a K-Alpha™ + X-ray photoelectron spectrometry (XPS, ThermoFisher Scientific Inc., Waltham, MA USA). Field emission scanning electron microscopy (FESEM, S-4800, Hitachi Co., Tokyo, Japan) and atomic force microscopy (AFM, Bruker, Billerica, MA, USA) were used to observe membrane morphology. The wettability properties of the membranes were obtained using an interfacial tensiometer (PD-VP Model, Kyowa Interface Science Co., Ltd., Niiza-City, Saitama, Japan). Underwater oil contact angle was measured using an automatic contact angle meter (OCA15EC, Dataphysics, Riverside, CA, USA).

### 2.3. Fabrication of Hollow Fiber Membranes

Figure S1 illustrates the set-up for fabricating the hollow fiber membrane [29]. Nitrogen gas was used as the compressor. A polymer solution containing 12 wt% PAN and 12 wt% PVP was dissolved in NMP. After complete dissolution, the polymer solution was degassed at 70 °C. A dry/wet spinning method was used for hollow fiber fabrication. Table 1 provides the details of the hollow fiber’s spinning conditions and parameters. The bore liquid was composed of 80% water and 20% NMP with a flow rate of 1 mL/min. The coagulation bath was water. The coagulation temperature was controlled at 25 °C. The airgap was maintained at 60 cm. Afterwards, the prepared membranes were soaked in 4000 ppm of sodium hypochlorite at 25 °C for 1 h to degrade residual PVP. They were then immersed in water to purge residual sodium hypochlorite and the degraded PVP [30]. The water where the membranes were immersed was changed every 12 h until use.

### 2.4. Surface Modification with TA-FeIII

The fabricated PAN hollow fiber membrane was immersed in a 2 g/L tannic acid solution that was adjusted to pH 9 using 1 M NaOH solution. After soaking for 1 min, excess solution was removed from the surface. The membrane was then saturated with nitric acid, followed by immersion in 2 g/L iron ion solution. The reaction time varied from 1 to 3 min. Once the reaction was completed, water was used to remove the excess solution. The membrane was then stored in water until testing.

### 2.5. Preparation of Oil–Water Emulsion

An oil–water emulsion was prepared by mixing distilled water and diesel with a volume ratio of 99:1. A 0.05 g/L of surfactant SDS was also added prior to high-speed stirring (1200 rpm) for one (1) hour. The size of the diesel emulsion was about 2.4 μm (Figure S2).

### 2.6. Crossflow Filtration and Antifouling Test

The hollow fibers were immersed in 50 wt% glycerol–water solution for half a day and then placed in the air for another half-day for drying. This prevented the shrinkage of the membranes during drying. Figure S3 shows the hollow fiber module for filtration testing. Hollow fiber with an effective length of 21 cm was placed in the tube, where both ends were sealed using epoxy resin. Afterwards, it was immersed in water to remove the glycerol prior to the filtration test. 

Figure S4 illustrates the crossflow filtration setup, which was also used for the antifouling and reusability test. The hollow fiber module was placed on the setup. Pre-compaction of the membrane was performed for 1 h at 1 bar. Then, the pure water flux was determined before changing the feed with oil–water emulsion. Flux (*J*) and oil rejection (*R*) were calculated as follows:

(1) $$J=\frac{V}{At}\;\;\;\;\;\;$$

(2) $$R\;\left(\%\right)=\left(1-\frac{C_{p}}{C_{f}}\right)\times 100$$

where *A* is the membrane effective membrane area (m^2^), *V* is the volume (L) collected at time *t* (h), and *C_P_* and *C_f_* are the concentrations of the permeate and the feed solution, respectively, which were determined using a total organic carbon machine (Vario TOC select: TOC/TNb Analyzer, Elementar, Langenselbold, Germany).

We evaluated antifouling of the membranes using a diesel oil–water emulsion. Similarly, the membrane was pre-compacted at 1 bar for 1 h. Then, the emulsion was fed to the setup. Every 10 min, the flux was recorded. One cycle comprised 70 min. The membrane and the filtration setup were cleaned by feeding 1000 ppm SDS for 5 min. Afterwards, clean water was fed again to measure the water flux. The following equations were used to calculate the flux recovery ratio (FRR, %), decay ratio (DR, %), reversible decay ratio (DR_r_,%), and irreversible decay ratio (DR_ir_, %):

(3) $$\mathrm{FRR}\;\left(\%\right)=\left(\frac{J_{w,2}}{J_{w,1}}\right)\times 100\%$$

(4) $$\mathrm{DR}\;\left(\%\right)=\left(1-\frac{J_{p}}{J_{w,1}}\right)\times 100\%$$

(5) $${\mathrm{DR}}_{r}\;\left(\%\right)=\left(\frac{J_{w,2}-J_{p}}{J_{w,1}}\right)\times 100\%$$

(6) $${\mathrm{DR}}_{\mathrm{ir}}\;\left(\%\right)=\left(\frac{J_{w,1}-J_{w,2}}{J_{w,1}}\right)\times 100\%$$

where J_w,1_ and J_w,2_ are the initial water flux of the membrane and the water flux after cleaning, respectively, and J_p_ is the flux when the feed was oil–water emulsion.

### 2.7. Determination of Molecular Weight Cutoff and Pore Size Distribution

A similar setup from Figure S4 was used to determine the molecular weight curoff (MWCO) of the membranes. The purpose of determining the MWCO was to understand the change in pore size and determine its ability to reject certain molecules. Different molecular weights of polyethylene glycol or polyethylene oxide (PEG 1000, 4000, 10 k, 35 k, 100 k, PEO 600 k and PEO 1 M) PEG were fed separately into the crossflow filtration setup. The concentration of the feed was fixed at 1000 ppm. The concentration in permeate was measured using a total organic carbon machine (Vario TOC select: TOC/TNb Analyzer, Elementar). The rejection was calculated using equation 2 from the manuscript. The mean pore diameter, geometric mean pore diameter and geometric pore standard deviation were calculated similarly to the work with Yan, Chung and Santoso [31].

## 3. Results and Discussion

Tannic acid has many phenol groups. Every three gallic acids of tannic acid can be used as a ligand, which reacts with an iron ion (FeIII) to form a coordination bond with a stable octahedral complex [26]. The polyphenolic group of tannic acid contains catechol and pyrogallol; these groups easily adhere to PAN, forming a structure of covalent bonds and non-covalent bonds, increasing the stability of the coating layer and the substrate layer.

Surface chemical analysis found TA-FeIII complex on the surface of the PAN hollow fiber. However, using FTIR cannot give precise changes in the spectra of the membrane. Because the TA-Fe layer is very thin, its spectra overlapped with the PAN spectra. Herein, we present the chemical composition of the membranes fabricated with different reaction times of TA and FeIII (Table 2). After deposition of TA-FeIII on the PAN surface, after only a few minutes, the TA already quickly reacted with FeIII to form a thin layer. For pure PAN membrane, an O element was presented because of some PVP that remained on the pores of PAN. After coating with TA-FeIII, more O element was detected in the modified membrane because TA is abundant with hydroxyl groups. The longer the reaction time of TA with FeIII, the more Fe present on the surface, resulting in an increase in O/C on the surface.

**Figure 1:**
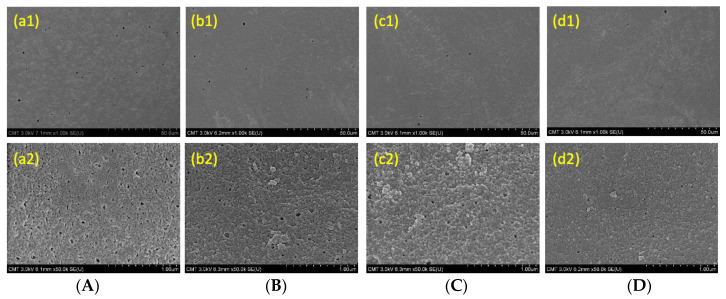
The outer surface SEM images of TA-FeIII/PAN membranes with different treatment time. (**A**) 0 min, (**B**) 1 min, (**C**) 2 min, (**D**) 3 min. Magnification (a1–d1) 1 K, (a2–d2) 50 K.

**Figure 2:**
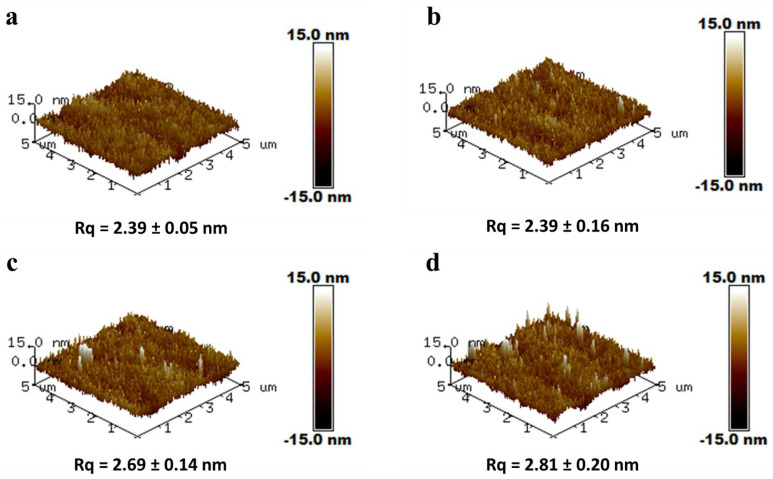
Three-dimensional AFM images of the membranes coated at different reaction times of TA and FeIII: (**a**) PAN; (**b**) 1 min; (**c**) 2 min; (**d**) 3 min; Lateral scale = 5 µm. Vertical scale = −15 to 15 nm.

Figure 1 presents the outer surface images of the membranes. Pristine PAN hollow fiber membranes contain numerous pores. When coated with TA-FeIII complex at different reaction times, these pores tend to become smaller and less common than in the pristine membrane. TA interacts with PAN through hydrogen bonding. Hence, its adhesion to PAN was strong enough not to peel off. More TA reacted with iron ions, which resulted in the complete growth of TA-FeIII; thus, the pores of PAN were covered. Amines were still detected in the XPS analysis in Table 2, indicating that the TA-FeIII coating layer was less than 10 nm, because the depth of analysis of the XPS was about 10 nm. Figure 2 shows the 3D AFM images of the membranes. The root mean square (Rq) represents the surface roughness of the membranes. When covered with TA-FeIII complex, the membrane became rougher because the presence of irons on the surface created a grainy structure [22]. Furthermore, FeIII induced aggregation of TA-FeIII complex on the PAN surface, thus increasing the surface roughness.

**Figure 3:**
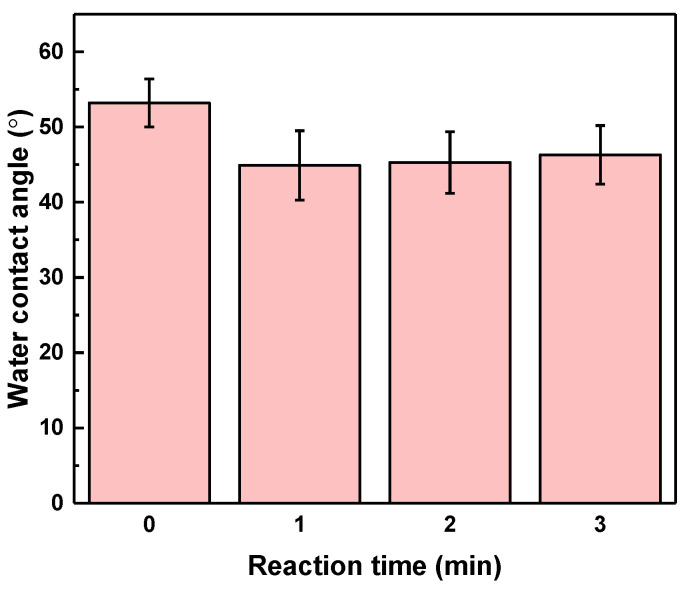
Water contact angle of the membranes, coated at different reaction times, of TA and FeIII.

A high surface roughness is also sometimes attributed to lower contact angle because of higher surface area. In addition, tannic acid has many polyphenolic groups, including hydrophilic quinone and glucinol. These hydrophilic groups make tannic acid a highly hydrophilic material. After the reaction of TA-FeIII on the surface of the PAN, the hydrophilicity of the membrane was expected to improve. Figure 3 indicates that the water contact angle of PAN is about 53.2°. When TA-FeIII complex covered the membrane, the water contact angle was reduced to 44.9°. However, after increasing the reaction time of TA-FeIII, the water contact angle remained similar. Nevertheless, coating with TA-FeIII enhanced the overall membrane hydrophilicity, which is beneficial for oil-in-water separation.

**Figure 4:**
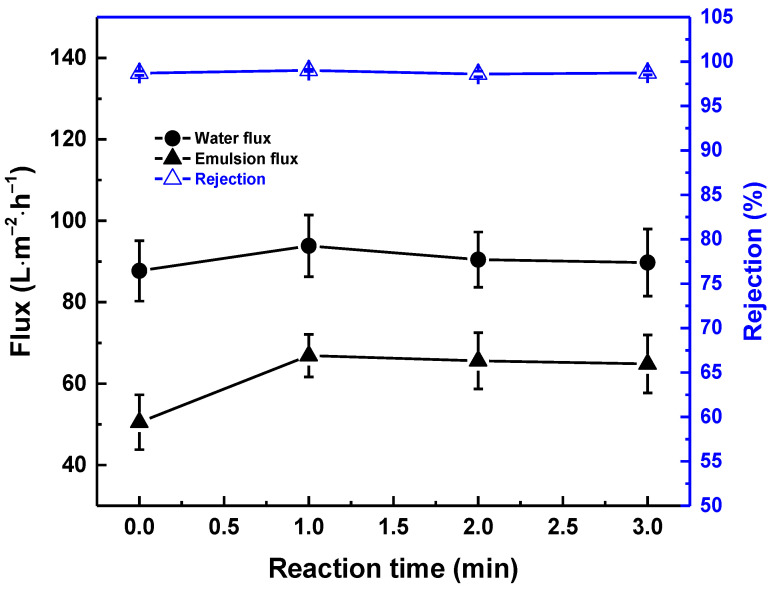
Effect of reaction time between TA and FeIII. Feed: H_2_O/diesel (99/1) emulsion at 1 bar.

**Figure 5:**
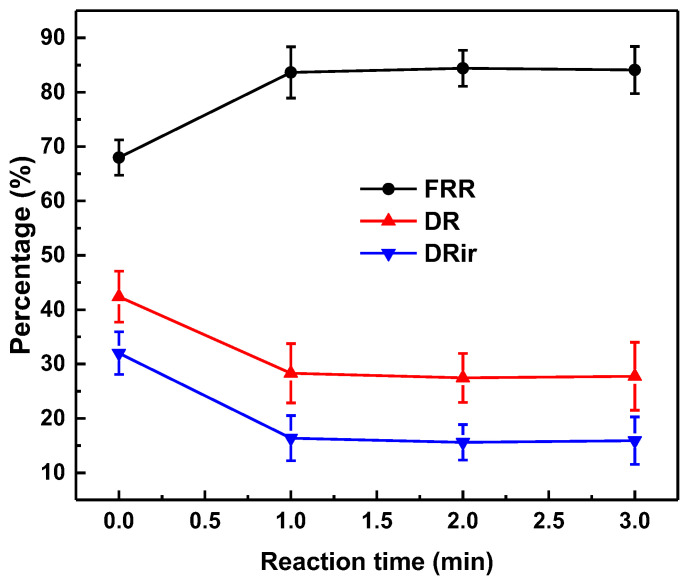
Effect of reaction time between TA and FeIII on antifouling performance. Feed: H_2_O/diesel (99/1) emulsion at 1 bar.

Figure 4 demonstrates that, after coating with TA-FeIII at different reaction times, the water flux and rejection remained similar. Even the hydrophilicity was enhanced; the coating contributes a little bit to increasing the overall mass transfer resistance. Thus, the water flux values of all the membranes were similar. However, when the feed was emulsion, the flux of the coated membrane reached up to 66 LMH. This increase in flux was because of the low adherence (Figure S5) of the oil onto the TA-FeIII surface, giving less mass transfer resistance for water. For all membrane conditions, the oil rejection was over 99%. Furthermore, the modified membranes had higher FRR (up to 80%) compared with pristine membrane (65%) (Figure 5). This shows that coating with TA-FeIII not only improves the flux of the membrane but also the antifouling property.

**Figure 6:**
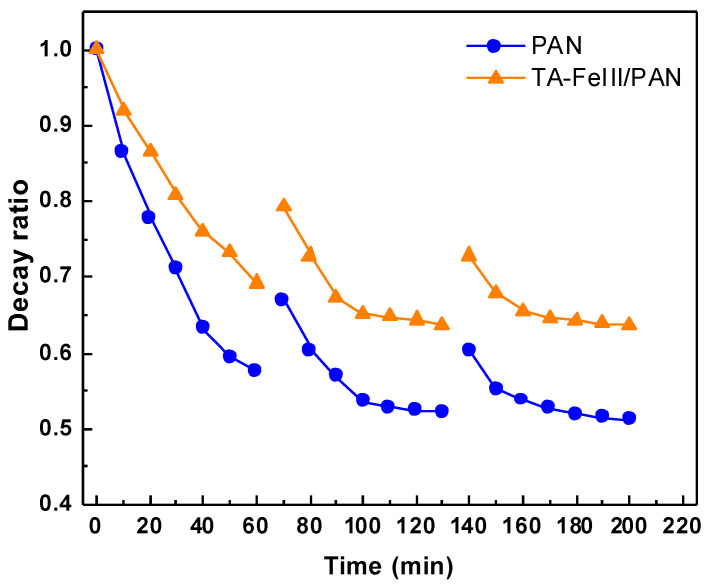
Reusability test of PAN and TA-FeIII membranes. Feed: H_2_O/diesel (99/1) emulsion at 1 bar.

Figure 5 and Figure 6 illustrate the antifouling properties and reusability of the membranes. Three cycles were performed to examine membrane reusability and antifouling properties. Membrane modified with TA-FeIII possessed higher FRR and low DR and DRir, indicating its better properties than those of pristine membrane. Pristine membrane had a FRR of below 70%, but modified membrane had a FRR value of over 80%. In the initial cycle, the pristine membrane instantly decreased in flux when it was fed with oil with a decay ratio of 0.55 at 60 min, whereas the modified membrane decreased only up to 0.70. After three cycles, the decay ratio of the pristine membrane decreased up to 0.5, whereas the modified membrane had a decayed ratio of 0.65 after 200 min of testing. The hydrophilicity of the TA-FeIII/PAN surface could easily release the attached foulant on the surface, thus creating a good antifouling property.

**Figure 7:**
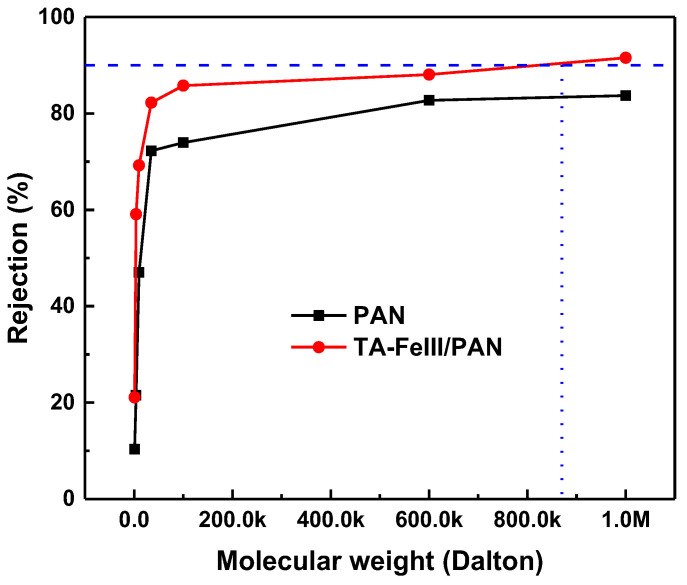
Molecular weight cutoff of the membranes.

Figure 7 and Table 3 show the molecular weight cutoff (MWCO) and pore diameter of the membrane. From the average molecular weight, the stokes radii of the solutes were calculated; hence, we also calculated the geometric mean pore diameter, which was described in previous work [31]. The modified membrane had a MWCO of 870.26 Da, which was relatively lower than PAN. Since the TA-FeIII covers the pores of PAN membrane and makes the membrane more hydrophilic, the decrease in geometric mean pore diameter of TA-FeIII (1.55 nm) did not affect the flux of the membranes. Furthermore, covering the pores of PAN using TA-FeIII not only increased the membrane hydrophilicity but also prevented the adhesion of the oils on the surface and on the pores. High underwater contact angle (Figure S5) evidences the low adhesion of oil on the modified membrane. The underwater contact angle was about 130°, indicating the membrane was oleophobic.

## 4. Conclusions

In this work, a PAN hollow fiber membrane was modified through deposition of TA-FeIII complex on its surface. The reaction of the TA with FeIII was varied from 0 to 3 min. TA interacts with PAN through hydrogen bonding. Increasing the reaction time, the surface became rougher due to the presence of FeIII on the surface. However, the contact angle of the modified membrane was similar at varying reaction times, but it was more hydrophilic than the pristine PAN membrane. In oil–water separation, the modified membrane shows better flux when fed by emulsion. At the optimum reaction time of 1 min, the modified membrane had better antifouling and reusability properties, with an FRR of over 80%. Moreover, even if the mean pore diameter decreased after deposition of the TA-FeIII, the modified membrane still had better separation efficiency. Therefore, TA-FeIII/PAN hollow fiber membrane shows promising performance for oil–water separation.

## Figures and Tables

**Table 1:** Spinning conditions and parameters.

Spinning Condition	Setting
Bore liquid composition	80/20 H_2_O/NMP
External coagulant	Water
Coagulation temperature	25 °C
Air gap	60 cm
Bore liquid flow rate	1 mL/min
Dope extrusion pressure	10 bar
Spinneret diameter	OD/ID, 2.0/0.9 (mm)
Humidity/temperature	70%/25 °C

**Table 2:** Surface elemental composition of the membranes from XPS analysis.

Reaction Time (min)	C	N	O	Fe	O/C
0	71.36	19.75	8.89		0.12
1	70.70	17.19	11.99	0.25	0.16
2	68.53	13.22	17.80	0.46	0.26
3	61.17	5.42	32.68	0.75	0.53

**Table 3:** Molecular weight cutoff, and pore diameter of the membranes.

Membrane Condition	MWCO (kDa)	Geometric Mean Pore Diameter (nm), μ_p_ = μ_s_	Geometric Pore Standard Deviation, σ_p_ = σ_g_
PAN		4.38	5.25
TA-FeIII	870.26	1.55	7.45

## Data Availability

Available upon request.

## Supplementary Materials

The following supporting information can be downloaded at: https://www.mdpi.com/article/10.3390/membranes13030351/s1, Figure S1. Schematic diagram of hollow fiber spinning apparatus. Figure S2. Size of the diesel emulsion from DLS. Figure S3. Membrane module for filtration test. Figure S4. Schematic diagram for crossflow filtration test. Figure S5. Underwater oil contact angle of the membranes at different reaction time.
