# Supplementary material for: Using Tannic-Acid-Based Complex to Modify Polyacrylonitrile Hollow Fiber Membrane for Efficient Oil-In-Water Separation
Source: Membranes (Basel). 2023 Mar 18;13(3):351. doi: 10.3390/membranes13030351 (PMC10051258; doi:10.3390/membranes13030351)
Supplement: Supplementary file 1 [file membranes-13-00351-s001.zip › membranes-2253056-supplementary.pdf]

membranes

Supplementary material

Using Tannic-Acid-Based Complex To Modify Polyacrylonitrile Hollow Fiber Membrane for Efficient Oil-In-Water Separation

Micah Belle Marie Yap Ang, Wei-Lin Hsu, You-Syuan Wang, Hsin-Yu Kuo, Hui-An Tsai * and Kueir-Rarn Lee *

R&D Center for Membrane Technology and Department of Chemical Engineering, Chung Yuan Christian University, Taoyuan 32023, Taiwan; mbmyang@gmail.com (M.B.M.Y.A.); ccbcc17@gmail.com (W.-L.H.); jimmy0970479726@gmail.com (Y.-S.W.); ksywyl@gmail.com (H.-Y.K.)

* Correspondence: huian@cycu.edu.tw (H.-A.T.); krlee@cycu.edu.tw (K.-R.L.)

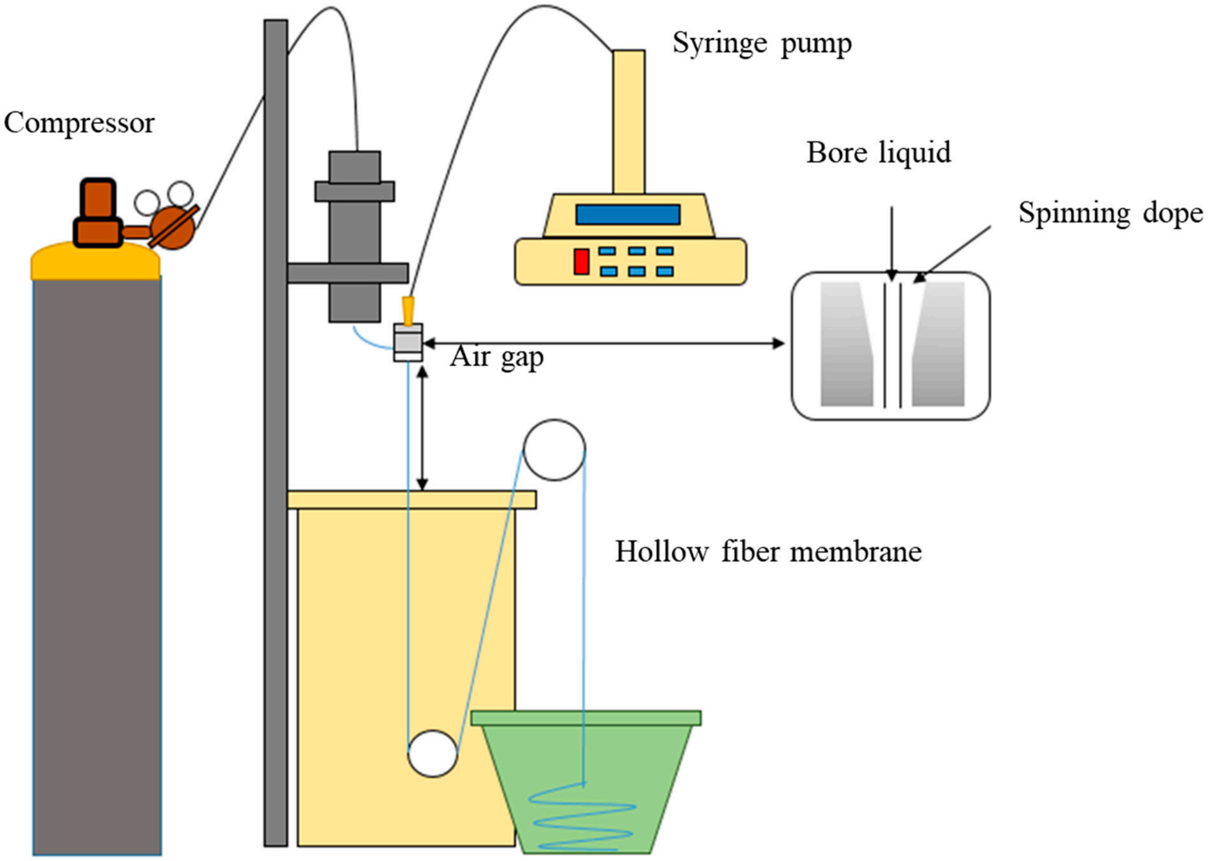

Figure S1. Schematic diagram of hollow fiber spinning apparatus.

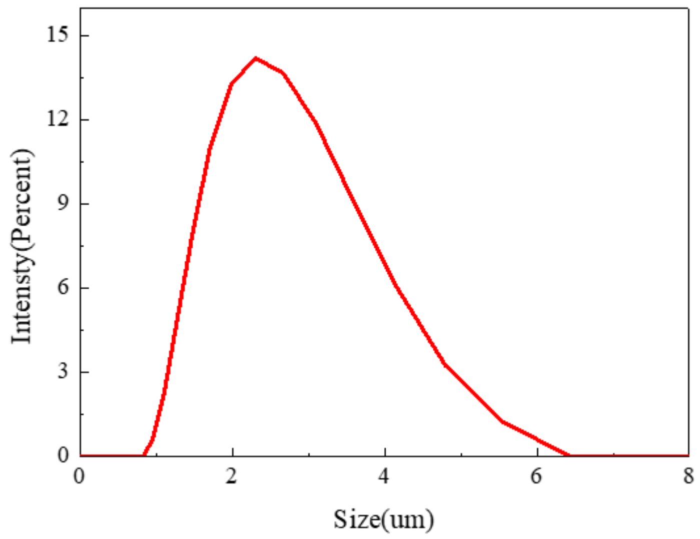

Figure S2. Size of the diesel emulsion from DLS.

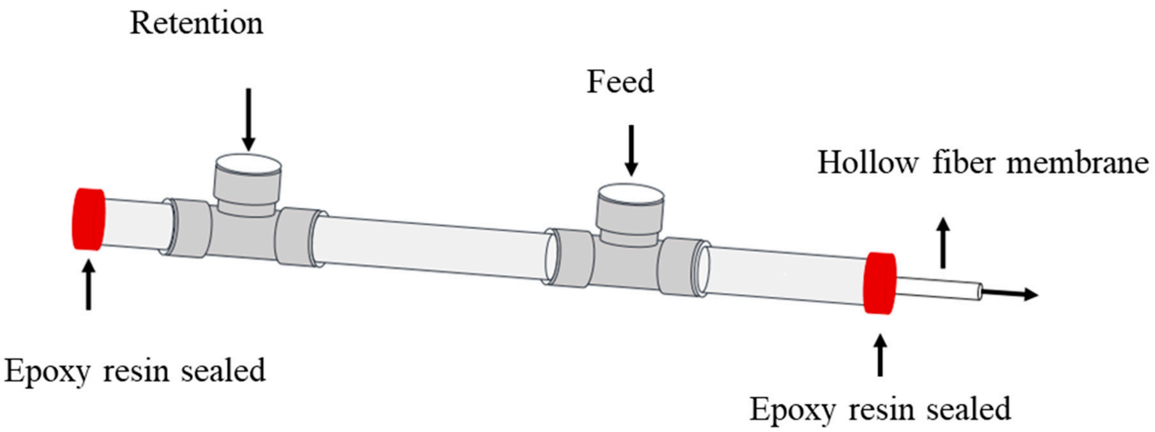

Figure S3. Membrane module for filtration test.

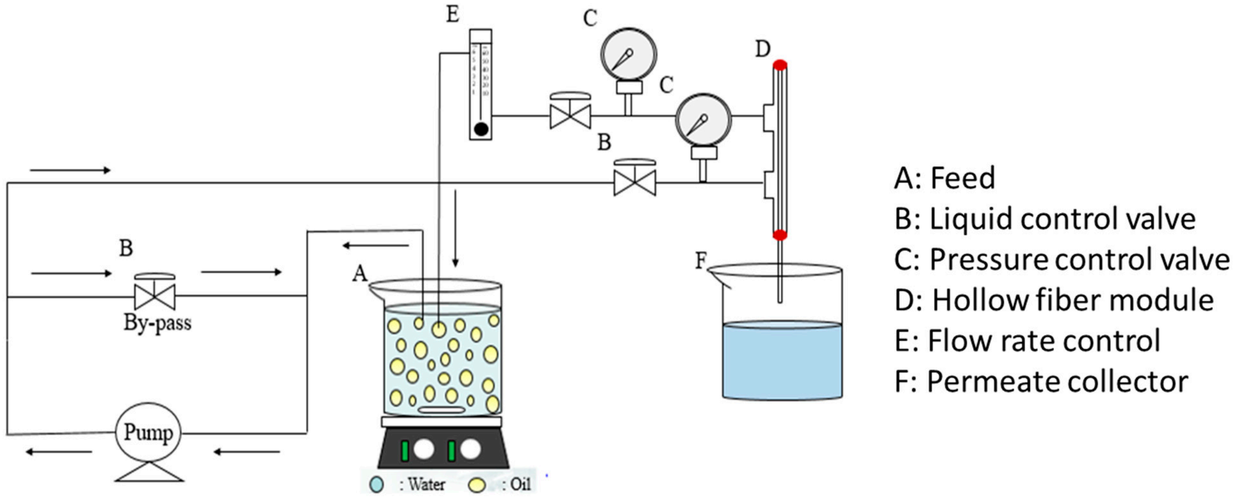

Figure S4. Schematic diagram for crossflow filtration test.

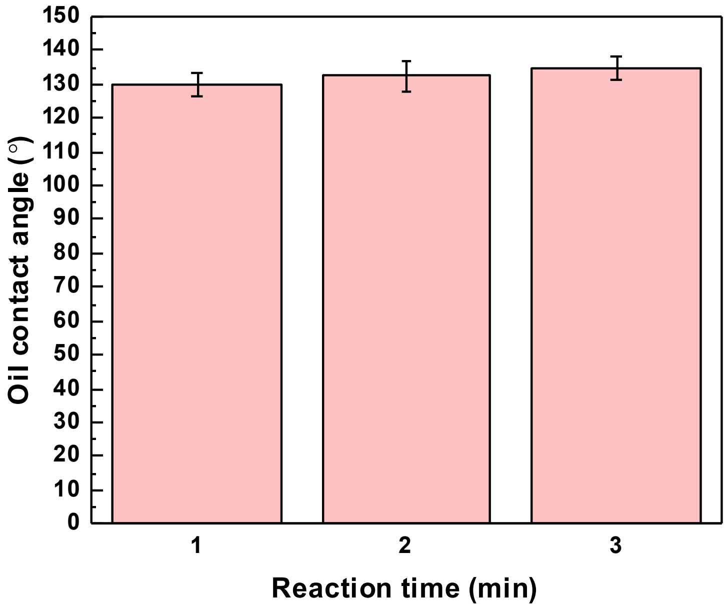

Figure S5. Underwater oil contact angle of the membranes at different reaction time.
